# Distinct roles for DNA-PK, ATM and ATR in RPA phosphorylation and checkpoint activation in response to replication stress

**DOI:** 10.1093/nar/gks849

**Published:** 2012-09-12

**Authors:** Shengqin Liu, Stephen O. Opiyo, Karoline Manthey, Jason G. Glanzer, Amanda K. Ashley, Courtney Amerin, Kyle Troksa, Meena Shrivastav, Jac A. Nickoloff, Greg G. Oakley

**Affiliations:** ^1^Department of Oral Biology, University of Nebraska Medical Center, Omaha, NE 68583, ^2^Department of Environmental and Radiological Health Sciences, Colorado State University, Fort Collins, CO 80523, ^3^Department of Molecular Genetics and Microbiology, University of New Mexico School of Medicine, Albuquerque, NM 87131 and ^4^Eppley Cancer Center, University of Nebraska Medical Center, Omaha, NE 68198, USA

## Abstract

DNA damage encountered by DNA replication forks poses risks of genome destabilization, a precursor to carcinogenesis. Damage checkpoint systems cause cell cycle arrest, promote repair and induce programed cell death when damage is severe. Checkpoints are critical parts of the DNA damage response network that act to suppress cancer. DNA damage and perturbation of replication machinery causes replication stress, characterized by accumulation of single-stranded DNA bound by replication protein A (RPA), which triggers activation of ataxia telangiectasia and Rad3 related (ATR) and phosphorylation of the RPA32, subunit of RPA, leading to Chk1 activation and arrest. DNA-dependent protein kinase catalytic subunit (DNA-PKcs) [a kinase related to ataxia telangiectasia mutated (ATM) and ATR] has well characterized roles in DNA double-strand break repair, but poorly understood roles in replication stress-induced RPA phosphorylation. We show that DNA-PKcs mutant cells fail to arrest replication following stress, and mutations in RPA32 phosphorylation sites targeted by DNA-PKcs increase the proportion of cells in mitosis, impair ATR signaling to Chk1 and confer a G2/M arrest defect. Inhibition of ATR and DNA-PK (but not ATM), mimic the defects observed in cells expressing mutant RPA32. Cells expressing mutant RPA32 or DNA-PKcs show sustained H2AX phosphorylation in response to replication stress that persists in cells entering mitosis, indicating inappropriate mitotic entry with unrepaired damage.

## INTRODUCTION

Cell division is regulated by intricate cell cycle control mechanisms that promote appropriate stepwise cell cycle progression, maintain genome integrity and suppress cancer. Cells respond to DNA damage by activating DNA repair and cell cycle checkpoint pathways. Cells are particularly vulnerable to DNA damage during S phase, which causes replication fork stalling or collapse, collectively called replication stress (1,2). Replication stress is also caused by topoisomerase and DNA polymerase poisons, and nucleotide pool depletion. If not restarted in a timely manner, stalled replication forks collapse to yield one-ended double-strand breaks (DSBs), or ‘double-strand ends’ (DSEs). Cells frequently experience replication stress at fragile sites (3) and DNA lesions caused by endogenous and exogenous sources, such as reactive oxygen/nitrogen species (4), genotoxic chemicals (5), ionizing radiation (6) and UV light (7). Many proteins involved in sensing, signaling and repairing DSBs also function in the replication stress response.

Cell cycle checkpoints require DNA damage sensors (e.g. MRE11), signal-transducers including phosphoinositol 3-kinase-related protein kinases (PIKKs), Chk1 and Chk2, and downstream effectors. These checkpoint systems amplify the damage signal and promote cell cycle arrest, DNA repair and cell survival (1,2,8). S phase checkpoints arrest ongoing replication, and prevent late origin firing, presumably to avoid fork stalling and collapse, but mutations in checkpoint proteins allow cells to progress through the cell cycle with damaged genomes, leading to genome rearrangements that promote cancer or mitotic catastrophe and cell death. Checkpoint proteins are cancer therapy targets, highlighting the importance of defining the proteins and mechanisms that regulate checkpoint pathways (9,10).

Ataxia telangiectasia mutated (ATM), ataxia telangiectasia and Rad3 related (ATR) and DNA-dependent protein kinase catalytic subunit (DNA-PKcs) are PIKKs with roles in checkpoint signaling and DNA repair. DNA-PKcs was originally defined by its role in DSB repair by non-homologous end-joining (NHEJ) but it also regulates proteins classically associated with homologous recombination (HR), including ATM, Werner protein (WRN) and others (11–15). Cells lacking DNA-PKcs display increased spontaneous HR (16), which is associated with replication problems at spontaneously arising DNA lesions (17). One PIKK target is replication protein A (RPA), the heterotrimeric single-stranded DNA (ssDNA) binding protein with critical roles in DNA replication and repair. RPA accumulates on long stretches of ssDNA at stalled and collapsed replication forks and is an important upstream signal for activation of the intra-S checkpoint (18). Previous studies revealed that DNA-PKcs and ATR phosphorylate the RPA32 subunit of RPA in response to replication stress (19,20), and that ATM and DNA-PKcs phosphorylate RPA32 in response to DSBs induced by ionizing radiation (21). Cell cycle arrest depends on PIKK-dependent phosphorylation/activation of upstream factors such as MRE11/RAD50/NBS1 (MRN), which interacts with phosphorylated RPA (22) and kinases including Chk1, which phosphorylate downstream targets that control cell cycle progression (23).

RPA32 is phosphorylated on multiple N-terminal residues during the cell cycle and in response to DNA damage. RPA32 Ser23 and Ser29 are fully phosphorylated during mitosis by cyclin-dependent kinase 1 (CDK1)/cyclin B (24,25) and partially phosphorylated by CDK2/cyclin A at the G1/S boundary (24,26,27). CDK phosphorylation of Ser23 and Ser29 is also induced during interphase by genotoxic stress (28,29), which stimulates phosphorylation of Ser33 by ATR that in turn promotes PIKK-mediated phosphorylation of residues closer to the N-terminus, including Thr21, Ser12, Ser4 and Ser8 (20,26,28–31). However, this is an overly simplistic model as there is evidence for distinct RPA32 phosphorylation pathways mediated by PIKKs with overlapping RPA32 target specificities that vary with replication stress agent and cell cycle phase. In addition, phosphorylation of certain RPA32 residues requires prior phosphorylation of other residues. For example, when cells are treated with camptothecin (CPT), blocking Ser33 phosphorylation with a Ser33Ala mutation suppresses Ser29 and Ser4/Ser8 phosphorylation, a Thr21Ala mutation suppresses Ser33, Ser29 and Ser4/Ser8 phosphorylation and a Ser23Ala mutation suppresses Ser33, Ser29, Thr21 and Ser4/Ser8 phosphorylation (29). These priming effects occur both in *cis* and in *trans* (29,31). Phosphorylated Ser4/Ser8 are found only in the most hyperphosphorylated form of RPA, among at least four phosphorylated forms. This suggests that phosphorylation of Ser4 and Ser8 are the final events in the maturation of DNA damaged-induced hyperphosphorylated RPA (30,32–34). Early studies revealed a role for DNA-PKcs in RPA32 phosphorylation in response to replication stress (19,28). Liaw *et al.* (35) recently reported that DNA-PKcs phosphorylation of RPA32 Ser4/Ser8 contributes to G2/M checkpoint arrest and HR suppression. Here, we extend these results by further defining PIKK roles in targeting specific RPA32 residues *in vitro* and *in vivo* in response to etoposide, identifying a novel reciprocal priming effect, and up- and downstream checkpoint signaling factors regulated by DNA-PKcs-mediated RPA32 phosphorylation and defining DNA-PKcs as a critical factor in both S and G2/M checkpoint control.

## MATERIALS AND METHODS

### Cell line construction and propagation

Chinese hamster ovary (CHO) V3 (DNA-PKcs defective) and derivatives complemented with wild-type (WT) or kinase-dead human DNA-PKcs were described and cultured as reported (16,36) in α-MEM (Invitrogen) supplemented with 10% fetal bovine serum, 100 U/ml penicillin and 100 µg/ml streptomycin. Human UM-SCC-38 oral squamous carcinoma cells, generously provided by T. Carey (University of Michigan), and its derivatives were cultured in Dulbecco's modified Eagle's medium (DMEM) (Invitrogen) supplemented as above.

Endogenous RPA32 in UM-SCC-38 cells was replaced with WT or S4A/S8A mutant RPA32, using retroviral vectors constructed as follows. A fragment encoding RPA32 amino acids 18–271 was amplified from a pET-11 d-RPA32 vector (kindly supplied by M. Wold, University of Iowa) using primers 5′-GCGCACCGGTGATATACATATGTGGAAC-3′ and 5′-CGCGGGATCCGTAAGCTCAGTAATCTGGAACATCGTATGGGTATTCTGCATCTGTGGA-3′ and digested with BamHI and NaeI, which adds a 3′ HA-tag. Double-stranded oligonucleotides, encoding amino acids 1–18 of WT or alanine-substituted Ser4/Ser8 RPA, were synthesized with AgeI and NaeI overhangs. The gene fragment and oligonucleotides were ligated into AgeI- or BamHI-digested pQCXIH, creating vectors for expression of WT and Ser4 → Ala/Ser8 → Ala (S4A/S8A) mutant RPA32. To create recombinant retrovirus, Phoenix cells (Orbigen) were seeded in 60-mm culture dishes at 5 × 10^6^ cells/ml, transfected with 24 µg of WT or S4A/S8A RPA vectors using Lipofectamine 2000 (Invitrogen), incubated for 48 h at 37°C and then 24 h at 32°C. The medium was collected, centrifuged (10 min at 2000(g) and added to 25% confluent UM-SCC-38 cells in the presence of 10 µg/ml polybrene (Sigma), incubated for 48 h, and transfected cells were selected with 20 µg/ml hygromycin (Sigma). After selection, the WT and S4A/S8A RPA32 expressing UM-SCC-38 cells were infected again, this time with supernatant from Phoenix cells transfected with a retroviral shRNA vector targeting the 3′-untranslated region of endogenous RPA32 (kindly supplied by X. Wu, Scripps Research Institute) and selected with 150 µg/ml G418. Human osteosarcoma U2OS cells expressing FLAG-wt-ATR (WT ATR) and FLAG-kd-ATR (kinase dead ATR) were gifts from P. Nghiem (University of Washington Medical Center).

### Induction of replication stress and PIKK inhibition

Cells were incubated in growth medium containing 20 µM etoposide for 2 h, then the medium was replaced with fresh medium and incubated for indicated times and harvested. Untreated cells are designated with a ‘−2’ time point. For the mitotic trap assay, cells were incubated with 0.3 µM nocodazole (Sigma) for 18 h before etoposide treatment. Replication stress was induced for indicated times with HU, *cis*-platin, CPT or etoposide at specified concentrations, then the medium was replaced and cells were harvested at various times after release from stress. For PIKK inhibition studies, cells were pre-treated for 3 h with 20 µM KU55933 (ATM inhibitor, ATMi) or 40 µM NU7026 (DNA-PKi) (EMD Biosciences), then incubated with 20 µM etoposide for 2 h in the presence of the inhibitor. After etoposide was removed, cells were incubated in fresh medium with the inhibitor until cells were harvested.

### Analysis of chromatin-associated proteins

UM-SCC-38 cells were harvested by centrifugation, washed in PBS and resuspended for 10 min on ice in cell lysis buffer [50 mM Tris–HCl, (pH 7.5), 150 mM NaCl, 0.1% Nonidet P-40, 10 mM NaF, 10 mM β-glycerophosphate, 1 mM Na_3_VO_4_ and protease inhibitor cocktail; Calbiochem] containing 0.5% Triton X-100. Chromatin fractionation was performed as previously described with slight modifications (37). Briefly, cells were washed once with PBS and incubated on ice in buffer A [10 mM HEPES, (pH 7.9), 10 mM KCl, 1.5 mM MgCl_2_, 0.34 M sucrose, 10% glycerol, 1 mM DTT, 10 mM NaF, 10 mM β-glycerophosphate, 1 mM Na_3_VO_4_ and phosphatase inhibitor cocktail] with 0.1% Triton X-100 for 5 min. Nuclei were collected by centrifugation (10 min, 1300*g*, 4°C) and washed once with buffer A and lysed in buffer B (3 mM EDTA, 0.2 mM EGTA, 1 mM DTT, 10 mM NaF, 10 mM β-glycerophosphate, 1 mM Na_3_VO_4_ and phosphatase inhibitor cocktail) for 30 min on ice. Insoluble chromatin was collected by centrifugation (5 min, 1700g, 4°C). The final chromatin pellet was resuspended in sodium dodecyl sulphate (SDS) sample buffer, boiled and chromatin-associated proteins were separated by SDS–polyacrylamide gel electrophoresis (SDS–PAGE).

### Immunoprecipitation, kinase reactions and protein detection by western blotting

Immunoprecipitation conditions for FLAG-tagged ATR (FLAG ATR) were adjusted to allow for co-precipitation of ATRIP with FLAG-ATR (38). FLAG-wild type-ATR (FLAG-wt-ATR) and FLAG-kinase dead-ATR (FLAG-kd-ATR) were induced in U2OS cells by culturing with 1.5 µg/ml doxycycline for 2 days (39). After induction, cells were washed with PBS, resuspended in cell lysis buffer for 30 min on ice and centrifuged for 20 min at 20 000*g*. The supernatants were incubated with anti-Flag M2 affinity gel (Sigma) at 4°C overnight. The FLAG-wt-ATR and FLAG-kd-ATR bound beads were washed three times with kinase wash buffer [20 mM HEPES (pH 7.4), 10 mM MgCl_2_, 2 mM DTT] and added immediately to kinase reactions performed by incubating 10 U of DNA-PKcs/Ku (Promega), immunoprecipitated FLAG-wt-ATR, or FLAG-kd-ATR at 37°C for 30 min in 30 µl of kinase buffer of 20 mM HEPES (pH 7.4), 10 mM MgCl_2_, 100 µM ATP, 2 mM DTT, 0.2 µg of sonicated salmon sperm DNA (Invitrogen), 0.5 µg of purified RPA and 10 µCi of [γ-^32^P]-ATP as indicated. Recombinant human RPA was purified as previously described (30). Kinase reactions were stopped by the addition of 1× Laemmli sample loading buffer. Proteins from these kinase reactions, whole cell lysates or immunoprecipitations were separated by SDS–PAGE, blotted onto polyvinylidene fluoride membranes, and probed with primary antibodies to RPA32 (ThermoFisher Scientific), p-T21-RPA32, p-S317-Chk1, β-actin (Abcam), p-S4/S8-RPA32, p-S33-RPA32, G6PD, ATR, nucleolin (Bethyl Laboratories), Chk1 G4, Ku70 (Santa Cruz Biotechnology), ATR or p-S345-Chk1 (Cell Signaling Technology), followed by Alexa Fluor 680-conjugated anti-rabbit (Invitrogen) and DyLight 800-conjugated anti-mouse (ThermoFisher Scientific) secondary antibodies, or horseradish peroxidase-conjugated antibodies (GE Healthcare). Images were obtained with a Typhoon 9410, or an Odyssey Imager, which detects specific phosphorylated forms of RPA32 and total RPA32 (regardless of phosphorylation status) simultaneously on a single membrane.

### Immunofluorescence microscopy

Cells were grown on four-well chamber slides or coverslips overnight prior to drug treatment, fixed with 100% ice-cold methanol for 10 min, permeabilized in 0.1% Triton X-100, washed and blocked in 1% milk in PBS for 30 min at room temperature and primary antibodies to α-tubulin, p-S139-H2AX or p-S-H3 (Santa Cruz Biotechnology) were applied in blocking solution for 1 h at room temperature, then an appropriate Alexa Fluor 488 or Alexa Fluor 568 conjugated secondary antibody (Invitrogen) was incubated in blocking solution for 1 h at room temperature. Cells were mounted in PermaFluor (Fisher) supplemented with 0.5 µg/ml 4′,6-diamidino-2-phenylindole (DAPI) (Roche). Images were captured digitally with a Zeiss Axiovert 200 M microscope, randomized and scored blindly. γ-H2AX was scored qualitatively (percent γ-H2AX positive cells) by visual examination, or quantitatively using ImageJ software as follows. DAPI positive regions in 20–30 nuclei were defined as regions of interest (ROI) per field, and five fields were scored per cell type, per condition. DAPI and γ-H2AX signals in each ROI were determined in blue and red channels, respectively. Background signals were measured in three non-DAPI regions, and the average background values were subtracted from nuclei values in both DAPI and γ-H2AX channels. Data collected included average γ-H2AX and DAPI signal intensity per nucleus, and average area of the ROI, giving quantitative estimates of DNA damage, DNA content, and size of nuclei, respectively.

### Flow cytometry

Cell cycle progression, histone H3 and γ-H2AX were monitored in UM-SCC-38 derivatives after etoposide treatment, incubation for indicated times and fixation in 70% ethanol overnight. Cells were permeabilized (0.25% Triton X-100 on ice for 15 min), washed, incubated overnight in PBS containing 0.1% BSA and p-S10-H3 or p-S139-H2AX antibodies (Millipore), washed and incubated in goat anti-mouse Alexa Fluor 647 antibody for 30 min at room temperature. Cells were incubated in 50 µg/ml propidium iodide and 100 µg/ml RNase A for 30 min, and 10 000 cells per sample were analyzed on a BD FACSarray (BD Biosciences) using 532 and 635 nm excitations and collecting fluorescent emissions with filters at 585/42 nm and 661/16 nm (yellow and red parameters, respectively). BD FACSarray and WinList™ (Verity House) software were used for data collection and analysis, respectively. BrdU incorporation was analyzed following cell growth in six-well culture dishes and treatment with 5 mM hydroxyurea (HU) or PBS control in growth medium for 1 h at 37°C. After three PBS washes, 100 μM BrdU was added in fresh growth medium. Samples were harvested at various times following HU release, fixed and processed with the FITC BrdU Flow Kit (BD Biosciences) according to manufacturer’s directions. Cells were analyzed with a MoFlo flow cytometer (Beckman Coulter) using 488-nm excitation, and emissions were collected 530/30 nm and 585/42 nm filters for FL-1 and FL-2 parameters, respectively. Summit software (Dako) was used for data collection and analysis.

## RESULTS

### PIKK phosphorylation of RPA32 after replication stress

There is general agreement in the literature that RPA32 Ser23 and Ser29 are CDK targets, and the remaining N-terminal sites (Ser4, Ser8, Ser11/12/13, Thr21 and Ser33) are phosphorylated by PIKKs, but there are discrepancies with respect to which PIKKs target specific sites. These discrepancies may reflect differences in genotoxic treatments, priming effects (29,31), lack of specificity of early PIKK inhibitors (e.g. wortmannin), DNA-PKcs-dependent expression of ATM (36,40–42) and other factors. For example, studies with DNA-PKcs mutant human cells, M059J, are difficult to interpret because these cells express ATM at low levels and the DNA-PKcs proficient M059K cells typically used as comparators are poorly matched (43). To clarify the roles of PIKKs and specific RPA32 phosphorylation sites in replication stress responses, we used RPA32 phospho-specific antibodies, PIKK inhibitors, isogenic human cell lines expressing WT or mutant RPA32 lacking Ser4/Ser8 phosphorylation sites and isogenic CHO cell lines expressing WT, null or kinase-dead DNA-PKcs. The kinase-dead DNA-PKcs cells are useful to compare with DNA-PKcs null cells because both lack DNA-PKcs kinase activity, but unlike null cells that express ATM at low levels, ATM is expressed at normal levels in DNA-PKcs kinase-dead cells (36).

**Figure 1.**
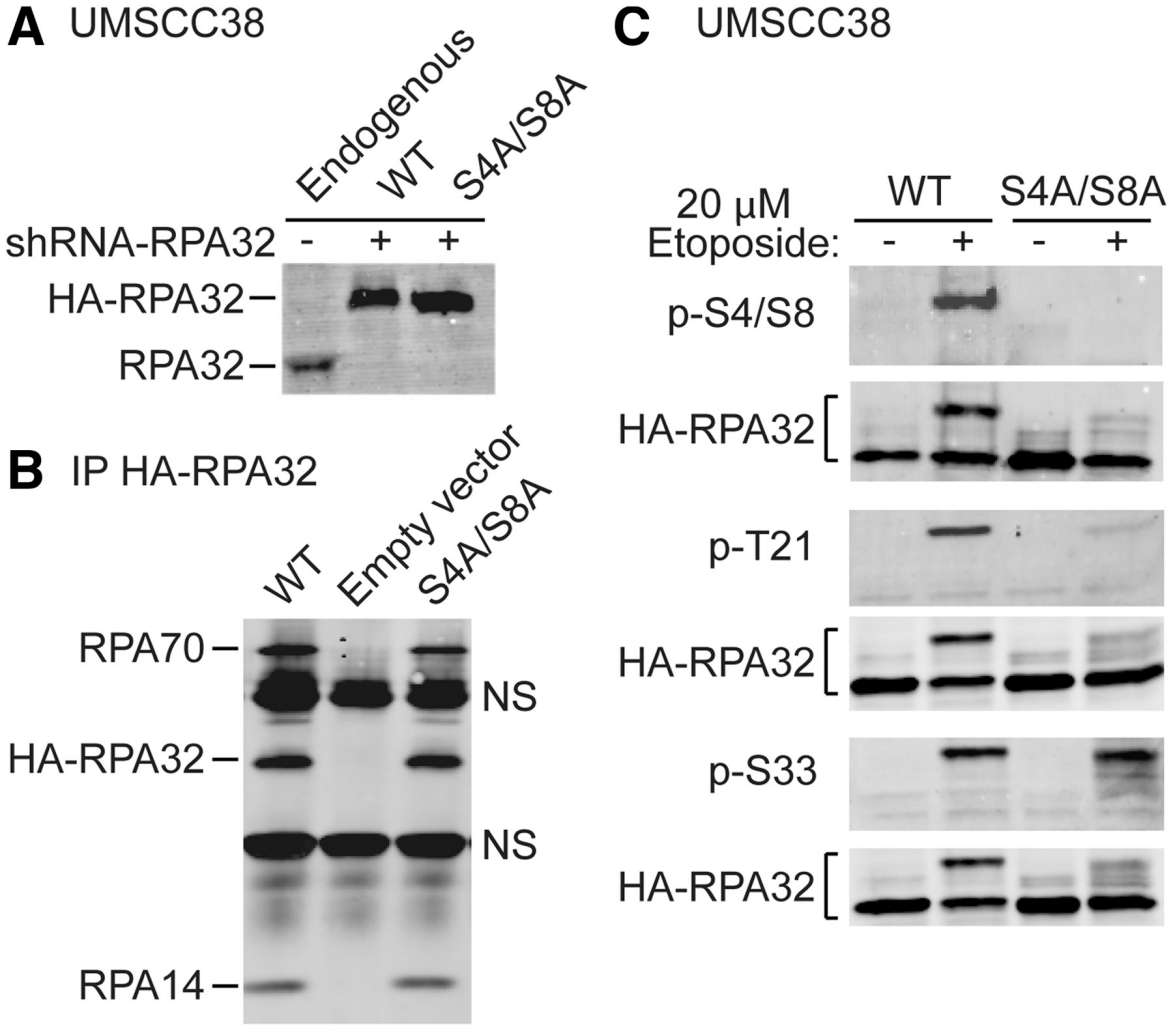
Etoposide-induced phosphorylation of RPA32 Thr21 requires Ser4/Ser8 phosphorylation. (**A**) Replacement of endogenous RPA32 with HA-tagged WT or S4A/S8A RPA32 in UM-SCC-38, analyzed by western blot probed with native RPA32 antibody. (**B**) HA-tagged WT and S4A/S8A RPA32 form RPA complex with RPA14 and RPA70. Total cell lysates were immunoprecipitated with anti-HA antibody and immunoblotted with RPA70, RPA32 and RPA14 antibodies. (**C**) UM-SCC-38 cells expressing WT or S4A/S8A RPA32 were treated with 20 µM etoposide for 2 h, then 2 h later, the proteins were harvested and analyzed by western blot with indicated antibodies.

We replaced endogenous RPA32 in human UM-SCC-38 cells by siRNA knockdown and expression of siRNA-resistant HA-tagged WT or S4A/S8A RPA32 (Figure 1A). Both WT and S4A/S8A RPA32 interacted with the endogenous RPA14 and RPA70 as all three subunits were detected when immunoprecipitated with anti-HA antibodies (Figure 1B). WT HA-RPA32 was functional *in vivo* as cells expressing this protein were viable, and phosphorylation of Ser4/Ser8, Thr21 and Ser33 was normally induced by replication stress caused by etoposide (Figure. 1C). As expected, Ser4/Ser8 phosphorylation was not detected in S4A/S8A mutant cells (Figure 1C). However, etoposide-treated S4A/S8A mutant cells did show low levels of phosphorylated RPA32 species, seen as slower migrating species on western blots probed with antibodies to native RPA32. Anantha *et al.* (29) showed that a Thr21Ala mutant reduces Ser4/Ser8 phosphorylation to ∼40% of WT levels. We found that Thr21 phosphorylation is reduced to <10% of WT levels in the S4A/S8A mutant (Figure 1C). Thus, Ser4/Ser8 and Thr21 phosphorylation show reciprocal priming effects.

It was shown previously that RPA32 Ser33 is largely or exclusively phosphorylated by ATR after etoposide, that a Ser33Ala mutant causes ∼2-fold reductions in phosphorylation of Ser29 (CDK-dependent) and Ser4/Ser8, but there is no effect on Thr21 phosphorylation (29). We found that etoposide induces Ser33 phosphorylation normally in the S4A/S8A mutant (Figure 1C). Thus, Ser33 phosphorylation regulates Ser4/Ser8 phosphorylation, but not vice versa. In addition, although the S4A/S8A mutant markedly reduces Thr21 phosphorylation, it has no effect on Ser33 phosphorylation, thus the partial block of Ser4/Ser8 phosphorylation in the Ser33Ala mutant observed by Anantha *et al.* (29) does not block Thr21 phosphorylation as effectively as the S4A/S8A mutant. These results further clarify the complex priming effects of various RPA32 phosphorylation sites.

### Defining PIKK roles in RPA32 phosphorylation *in vitro* and *in vivo* after replication stress

**Figure 2.**
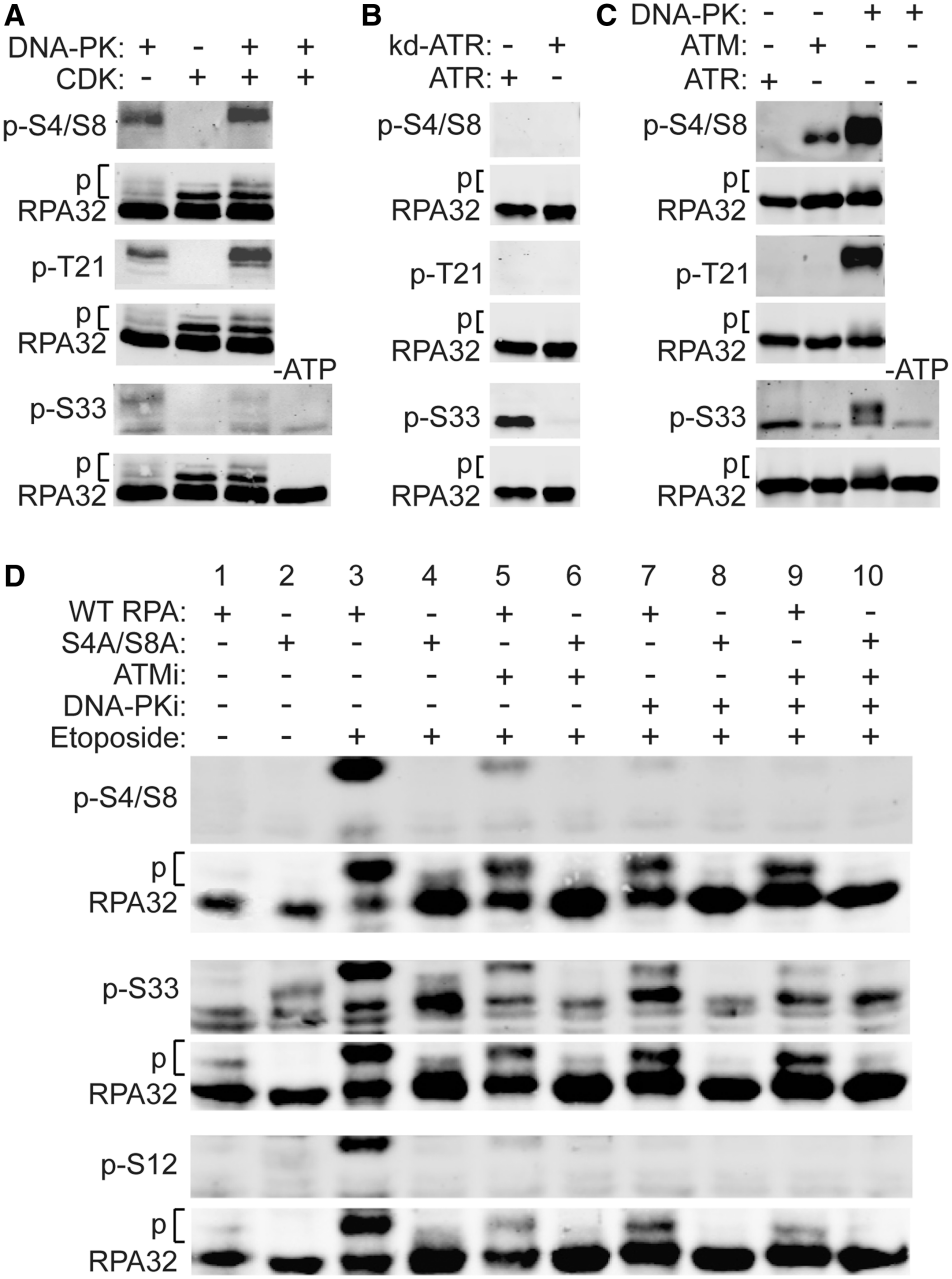
PIKK phosphorylation of RPA32. (**A–C**) *In vitro* phosphorylation of RPA32 with purified CDK, PIKKs or kinase-dead ATR, as indicated above each lane. Total RPA32 and phospho-specific forms were detected with indicated antibodies by western blot. Phosphorylated forms migrate slower (indicated by ‘p’). In panels A and C, control reactions lacking ATP are shown. (**D**) UM-SCC-38 cells expressing WT or S4A/S8A RPA32 were treated for 2 h with 100 µM etoposide, and with 10 µM KU55933 (ATMi), 20 µM NU7026 (DNA-PKi) 1 h prior to etoposide and added back after etoposide removal or mock treated as indicated, whole cell extracts were prepared and RPA32, and specific phospho-forms, were detected by western blot using indicated antibodies.

*In vitro* kinase assays and western blots with phospho-specific antibodies were used to further clarify PIKK roles in phosphorylating specific RPA32 residues. In cells treated with CPT, CDK phosphorylation of RPA32 Ser23 and Ser29 primes Thr21, Ser4 and Ser8 phosphorylation by DNA-PK (29). We therefore, incubated RPA32 with DNA-PK alone and in combination with CDK. RPA32 Ser4/Ser8 and Thr21 were phosphorylated by DNA-PK (Figure 2A–C), but not CDK, although the addition of CDK significantly enhanced phosphorylation of Thr21, consistent with *in vivo* results of Anantha *et al.* (29). CDK also slightly enhanced phosphorylation of Ser4/Ser8 (Figure 2A), consistent with *in vitro* results of Pan *et al.* (44). Ser4/Ser8 are not consensus PIKK target sequences, yet previous studies implicated DNA-PK in Ser4/Ser8 phosphorylation *in vitro* and *in vivo* (19,28,35,44); the data in Figure 2A is the first direct evidence, using phospho-specific antibodies, demonstrating DNA-PK targets Ser4/Se8 *in vitro*. DNA-PK also phosphorylated Ser33 (Figure 2A–C, most clearly seen in Figure 2C), but was far less efficient compared with DNA-PK phosphorylation of Ser4/Ser8 and Thr21 (Figure 2B). CDK phosphorylates Ser23 and Ser29 *in vivo* (29,45), and based on these previous results, our *in vitro* results, shown as slower migrating species on western blots probed with antibodies to native RPA32, most likely represent phosphorylation at Ser23 and Ser29 (Figure 2A). Interestingly, ATM phosphorylated Ser4/Ser8, but not Thr21, and ATM showed minimal activity toward Ser33 (Figure 2C). Controls confirmed that these reactions are ATP-dependent (Figure 2A and C, bottom panels).

We next investigated ATM and DNA-PK roles in etoposide-induced phosphorylation of RPA32 *in vivo* at Ser4/Ser8, Ser33 and the poorly characterized Ser12 site. UM-SCC-38 cells expressing WT or S4A/S8A mutant RPA32 were treated with 20 µM etoposide for 2 h in the presence or absence of the ATM inhibitor KU55933 (ATMi), the DNA-PK inhibitor NU7026 (DNA-PKi) or both (Figure 2D). Ser4/Ser8 were efficiently phosphorylated after etoposide and this signal was absent in S4A/S8A mutant cells. Ser4/Ser8 phosphorylation was reduced 7-fold by ATMi, 25-fold by DNA-PKi and 35-fold by both inhibitors, indicating that DNA-PK is the dominant kinase that phosphorylates Ser4/Ser8 *in vivo* in response to replication stress.

Ser33 phosphorylation was seen in WT cells in both hyperphosphorylated RPA32, and faster migrating (less phosphorylated) species. As expected (30,32–34), the hyperphosphorylated form was absent in S4A/S8A mutant cells. In WT cells, Ser33 phosphorylation was reduced 2.5-fold by ATMi, 1.4-fold by DNA-PKi and 2.5-fold by both inhibitors. Although ATMi and DNA-PKi modestly reduced Ser33 phosphorylation, much of the p-Ser33 signal migrated below the hyperphosphorylated species, as expected, because DNA-PKi and ATMi block Ser4/Ser8 phosphorylation. The residual Ser33 phosphorylation in the presence of ATMi and DNA-PKi is probably due to ATR (Figure 2B). When Ser4/Ser8 phosphorylation is completely blocked in the S4A/S8A mutant, Ser33 phosphorylation is reduced 1.4-fold without kinase inhibitors and ∼6-fold with either ATMi or DNA-PKi, thus Ser4/Ser8 phosphorylation (by ATM and DNA-PKcs) moderately enhances Ser33 phosphorylation by ATR.

RPA32 Ser12 is phosphorylated by DNA-PK *in vitro*, and is phosphorylated *in vivo* by unknown kinase(s) in response to replication stress (29,31). We found that Ser12 phosphorylation in response to etoposide was reduced by ∼25-fold by ATMi or DNA-PKi, and by ∼70-fold by both inhibitors. Interestingly, Ser12 phosphorylation is completely blocked in the S4A/S8A mutant, thus Ser4/Ser8 phosphorylation appears to be essential to prime Ser12 phosphorylation. DNA-PKi and ATMi block Ser4/Ser8 phosphorylation (Figure 2D), thus it is not possible to assign a PIKK to Ser12 because the reduction in Ser12 phosphorylation with ATMi and DNA-PKi could reflect a direct effect or an indirect effect of blocking Ser4/Ser8 phosphorylation.

### RPA32 Ser4/Ser8 phosphorylation regulates replication checkpoint signaling via MRE11 and TopBP1 phosphorylation

**Figure 3.**
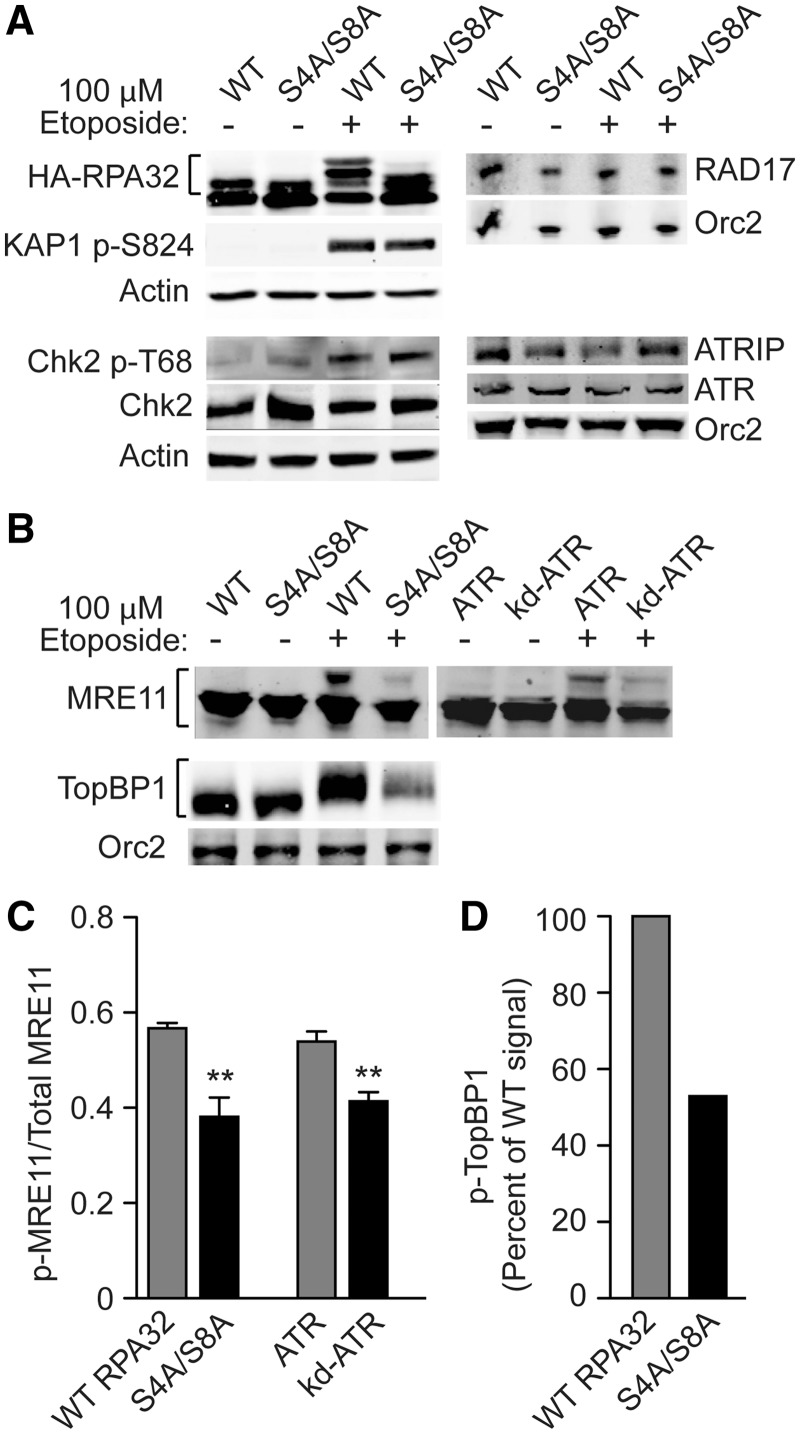
Phosphorylation of RPA32 Ser4/Ser8 regulates activation of a subset of upstream checkpoint proteins. (**A**) UM-SCC-38 cells expressing WT or S4A/S8A RPA32 were treated with etoposide as in Figure 2, and phosphorylation status of RPA32, KAP1 and Chk2, and total levels of RAD17, ATRIP and ATR were monitored by western blot. (**B**) Etoposide-induced phosphorylation of MRE11 and TopBP1 is sharply reduced in cells expressing S4A/S8A mutant RPA32. Cells expressing kinase-dead ATR serve as a control for blocked MRE11 phosphorylation. Orc2 and β-actin serve as loading controls in panels A and B. (**C**) Densitometric quantitation of phospho-MRE11. Average fractions (±S.E.M.) of etoposide-induced phospho-MRE11 relative to total (phospho- and non-phospho-) MRE11 signals (quantitated with ImageJ software) for three independent determinations in WT, S4A/S8A, and ATR-kd mutants (panel B shows a representative blot). **P < 0.01 (*t*-tests). (**D**) Quantitation of phospho-TopBP1 in panel B, normalized to Orc2 loading.

ATM, ATR and DNA-PK are key upstream damage response signaling kinases that regulate cell cycle progression, DNA repair, programed cell death and other systems. RPA bound to ssDNA, either at resected DSBs or stalled replication forks, recruits ATRIP-ATR leading to ATR activation and phosphorylation of downstream checkpoint proteins (18). It is known that hyperphosphorylated RPA32 is important for checkpoint activation, including delaying mitotic entry when DNA is damaged (34,35). To clarify the mechanism by which RPA32 hyperphosphorylation contributes to checkpoint activation, we monitored phosphorylation of several downstream PIKK targets in etoposide-treated cells expressing WT or S4A/S8A RPA32. As shown in Figure 3A, the S4A/S8A mutant showed normal phosphorylation/activation of Rad17 and Chk2 checkpoint proteins and KAP1, a protein phosphorylated by ATM that regulates access of DSB repair proteins to heterochromatin and transcription of the p21/CIP1 checkpoint effector (46).

We next investigated the MRN-ATR-Chk1 arm of the replication checkpoint. MRN functions in checkpoint activation both as an upstream damage sensor and a downstream target of ATR (47). We reported that MRN co-localizes with RPA in cells treated with UV, HU and etoposide, the MRN–RPA interaction is regulated by phosphorylation and dephosphorylation of these complexes, that an RPA–MRN interaction followed by RPA32 phosphorylation was required for etoposide-induced G2/M arrest, and that HU-induced phosphorylation of RPA32 by ATR requires NBS1 (22,48,49). NBS1 also has an important role in etoposide-induced, ATR-dependent RPA32 hyperphosphorylation (50) and PPA2-mediated RPA32 dephosphorylation of RPA32 Thr21 and Ser33 is required for checkpoint release and cell cycle re-entry (51). We found that etoposide-induced MRE11 phosphorylation was significantly reduced in UM-SCC-38 cells expressing S4A/S8A mutant RPA32 compared with WT, an effect comparable with that observed in cells expressing kinase-dead ATR (Figure 3B and C). Full ATR activation depends on its interaction with TopBP1 (52), and specifically on phosphorylation of PIKK consensus sites in TopBP1’s ATR activation domain (53,54). Thus, TopBP1 phosphorylation is important for replication checkpoint activation. Similar to MRE11, etoposide-induced TopBP1 phosphorylation was reduced in UM-SCC-38 cells to ∼50% of WT levels in the S4A/S8A mutant (Figure 3B and C). These results indicate that RPA32 Ser4/Ser8 phosphorylation is a critical early step for full ATR activation in response to replication stress and subsequent replication checkpoint arrest.

### Replication-induced Chk1 phosphorylation is defective in DNA-PKcs and RPA32 Ser4/Ser8 mutants

**Figure 4.**
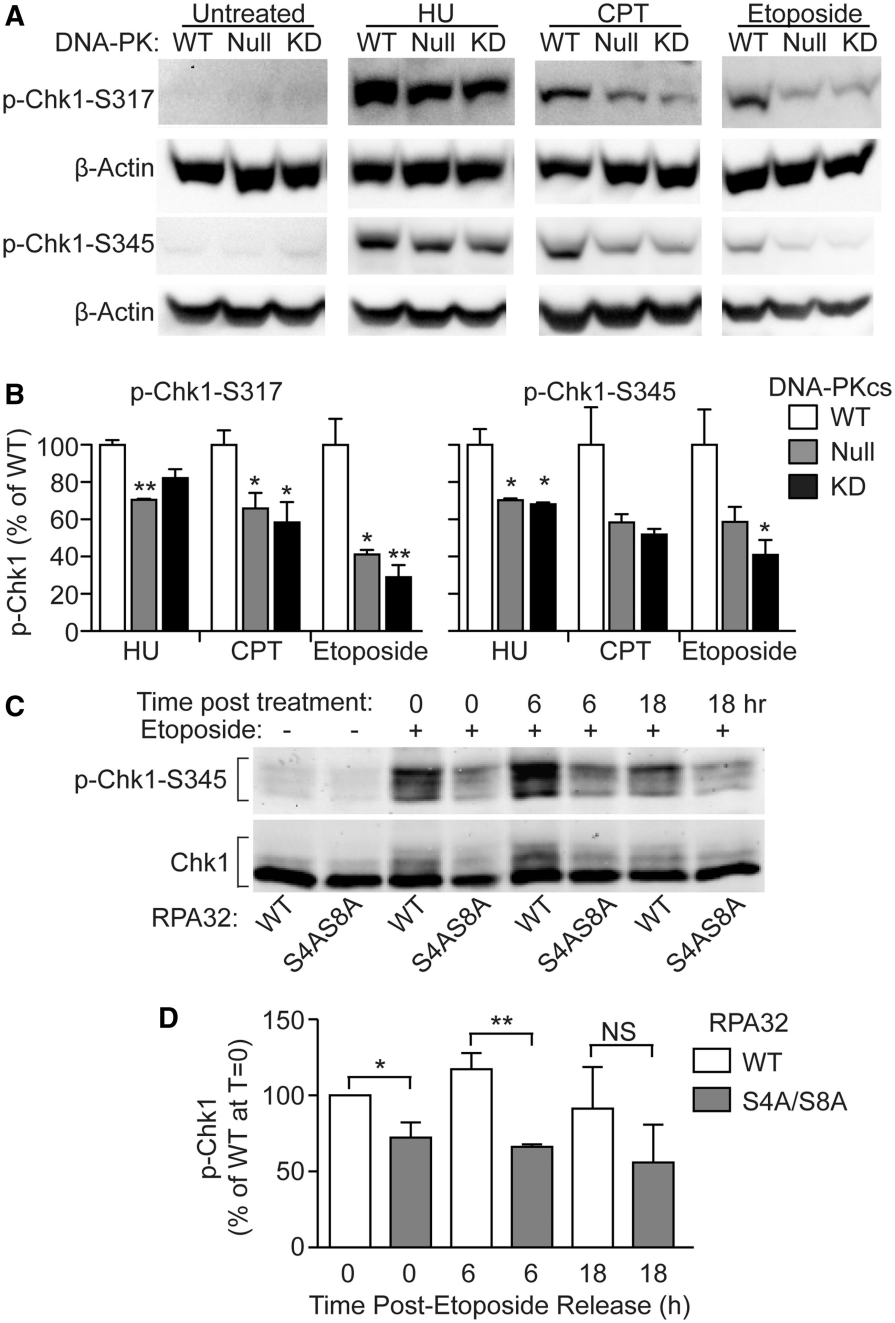
DNA-PKcs phosphorylation of RPA32 Ser4/Ser8 regulates Chk1 activation. (**A**) CHO cells lacking DNA-PKcs or complemented with WT or kinase-dead DNA-PKcs were treated for 30 min with 10 µM CPT or 100 mM HU for 1 h with 100 µM etoposide or untreated, and Chk1 Ser317 phosphorylation was monitored by western blot; representative blots of two to three independent determinations are shown). β-Actin serves as the loading control. (**B**) Quantitation of Chk1 phospho-Ser317 and phospho-Ser345 induction with HU, CPT or etoposide treatment versus untreated cells. Values are average p-Chk1 signals (±S.E.M.) of two to three independent determinations normalized to β-actin loading and shown as percentage of WT levels. *P < 0.05; **P < 0.01 (*t*-tests). (**C**) Phosphorylation of Chk1 Ser345 and Chk1 were monitored with respective antibodies in UM-SCC-38 cells expressing WT or S4A/S8A RPA32 treated (or mock treated) with 20 µM etoposide for 2 h and released for 0, 6 or 18 h; representative blots of three independent determinations are shown. (**D**) Quantitation of p-Chk1 induction in panel C as described for panel B, except p-Chk1 signals were normalized to total Chk1 levels and shown as percentages of the WT signal immediately after etoposide treatment (*T* = 0).

Replication stress activates ATR, which phosphorylates/activates Chk1, and this is a key step in transmitting DNA damage signals to downstream replication checkpoint effector proteins (52,55). Having established DNA-PKcs as the major PIKK targeting RPA32 Ser4/Ser8 (Figure 2), and that Ser4/Ser8 phosphorylation (through MRE11 and TopBP1 signaling, Figure 3) is important for full ATR activation, we next monitored Chk1 phosphorylation in cells with DNA-PKcs defects or expressing S4A/S8A mutant RPA32. DNA-PKcs null and kinase dead mutant CHO cells showed a marked reduction in Chk1 phosphorylation in response to HU, CPT or etoposide (Figure 4A and B), and a similar but more muted defect was seen in etoposide-treated UM-SCC-38 cells expressing RPA32 S4A/S8A (Figure 4C and D). These results suggest that DNA-PKcs phosphorylation of Ser4/Ser8 contributes to Chk1 activation and DNA-PKcs also contributes to Chk1 activation through mechanisms independent of Ser4/Ser8 phosphorylation.

### RPA32 Ser12 phosphorylation correlates with replication recovery after replication stress

**Figure 5.**
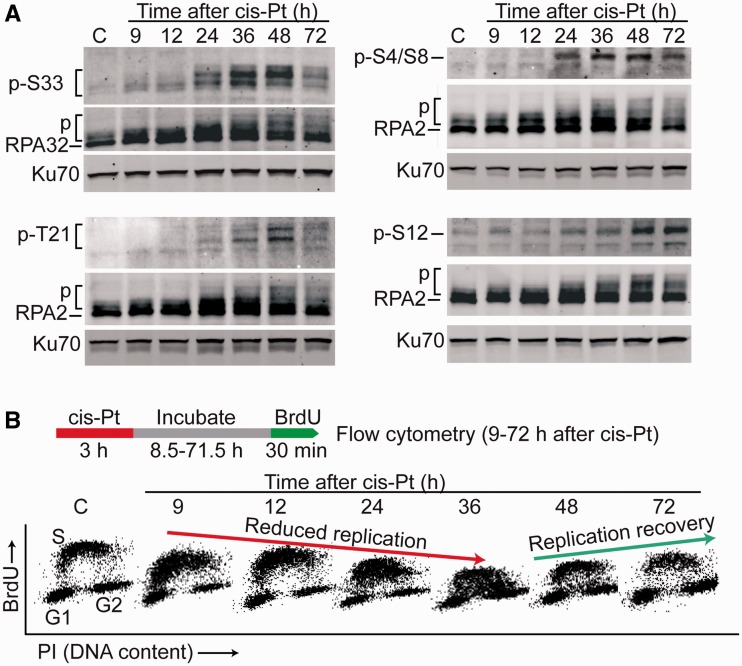
Specific RPA32 phosphorylation events correlate with replication arrest and recovery after *cis*-platin treatment and release. (**A**) Specific phospho-RPA32 forms were analyzed as in Figure 1A in UM-SCC-38 cells after 3 h, 20-µM *cis*-platin treatment and release for indicated times. (**B**) Using the same treatment scheme and cell harvest time points in panel A, cells were additionally pulsed with BrdU for 30 min, then BrdU incorporation into DNA and total DNA content (PI) were monitored by flow cytometry. BrdU signals decrease over a 36-h period after release from *cis*-platin, and then increase between 48 and 72 h.

Damaged cells will undergo cell cycle arrest, but eventually, the checkpoint is abrogated and cells re-enter the cell cycle. These events can be monitored in S phase as replication arrest and subsequent recovery by nucleotide incorporation assays. To further clarify the role of specific RPA32 phosphorylation events in replication arrest and recovery, we investigated the kinetics of RPA32 Ser4/Ser8, Thr21, Ser33 and Ser12 phosphorylation in UM-SCC-38 cells for 9–72 h after release from a 3 h *cis*-platin treatment, and correlated these data with BrdU incorporation monitored by flow cytometry. As shown in Figure 5A, Ser4/Ser8, Thr21 and Ser33 showed maximum phosphorylation from 24 to 48 h after release from *cis*-platin (Figure 5A), correlating with replication suppression (Figure 5B). These residues were dephosphorylated at later times, consistent with prior results showing Thr21 and Ser33 dephosphorylation is required for replication recovery (51). In contrast, Ser12 was not phosphorylated at early times, but was phosphorylated at 48–72 h after *cis*-platin release, when replication resumed. These results suggest Ser4/Ser8, Thr21 and Ser33 phosphorylation mark checkpoint arrest, and S12 phosphorylation marks replication recovery.

### DNA-PKcs phosphorylation of RPA32 Ser4/Ser8 promotes repair of replication-stress-induced DSBs

**Figure 6.**
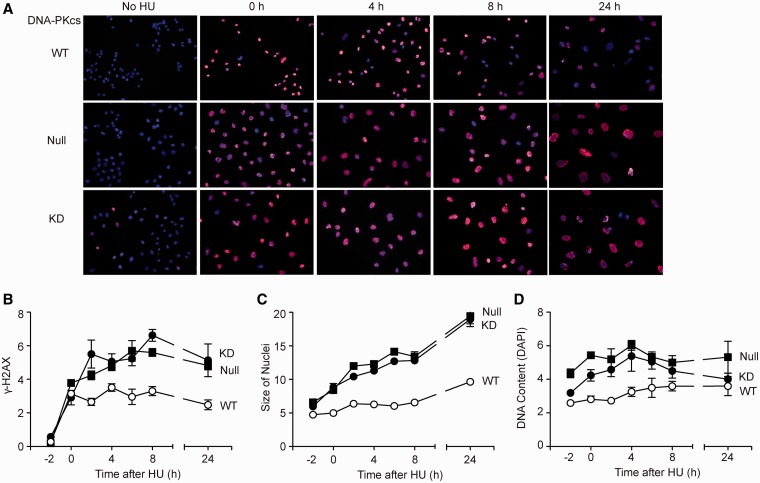
DNA-PK promotes (γ-H2AX resolution after release from HU. (**A**) Representative immunofluorescence images of WT, null and kinase-dead DNA-PKcs cells treated with 5 mM HU for 16 h and released for indicated times; (γ-H2AX, red; DAPI, blue. (**B–D**) Quantiation of panel A including average (γ-H2AX signal, nuclear size and DNA content (DAPI) based on analysis of 5 fields with an average of 20 or more cells per field per condition, using ImageJ software.

**Figure 7.**
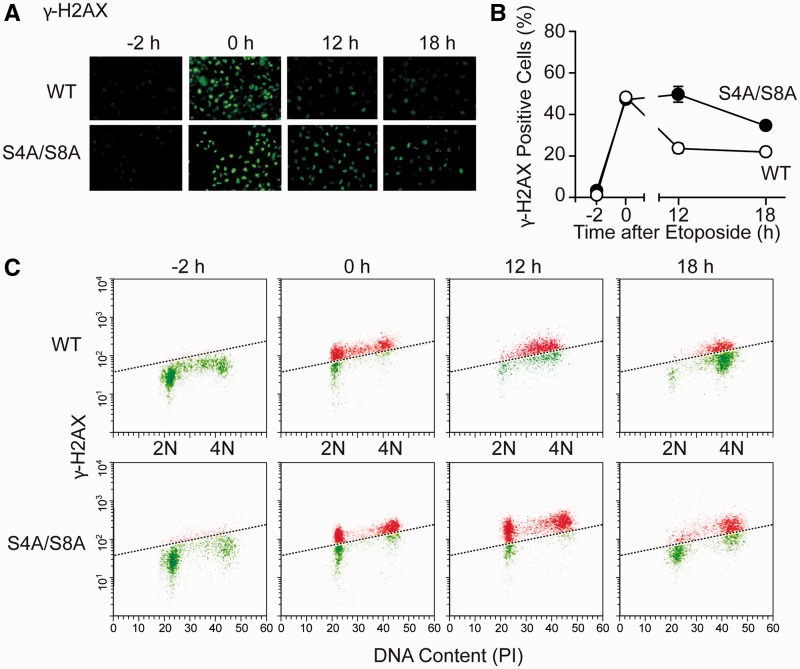
Phosphorylation of RPA32 Ser4/Ser8 promotes γ-H2AX resolution after release from etoposide. (**A**) γ-H2AX analysis as in Figure 6A in UM-SCC-38 cells expressing WT or S4A/S8A RPA32 treated with etoposide for 2 h and released for indicated times. (**B**) Quantitation of panel A, average (±S.E.M.) γ-H2AX positive cells based on five fields with 20–30 cells scored per field for three independent experiments. (**C**) Flow cytometric analysis of γ-H2AX and DNA content measured over the same treatment course in panels A and B. γ-H2AX positive cells, above the dashed line, are more abundant in the S4A/S8A mutant at 12 and 18 h after etoposide release.

The replication checkpoint defect of cells with DNA-PKcs defects or S4A/S8A RPA32 suggests that these mutants progress through the cell cycle while still harboring unresolved DNA lesions. To explore the consequences of checkpoint failure in these mutants, we first examined induction and resolution of γ-H2AX in DNA-PKcs defective CHO cells treated with HU and released. The level of γ-H2AX is a reasonable measure of DSBs and sites of excessive ssDNA at stalled forks (56,57) and therefore, can be used to monitor replication stress-induced damage and subsequent repair. As mammalian cells activate as many as 500 origins (1000 forks) at a time during S phase (58), individual γ-H2AX foci are not visible. We analyzed γ-H2AX immunofluorescence signals using ImageJ software in an average of 126 DAPI-stained nuclei per treatment group at each time point in five independent microscopy fields (Figure 6A). DNA-PKcs status did not affect the initial induction of γ-H2AX by HU, but γ-H2AX levels continued to increase in both DNA-PKcs null and KD mutants for several hours after HU release and remained higher than WT over the 24 h time course (Figure 6B). After HU treatment and release, we observed a slight increase in the average size of WT nuclei (measured using the area function of ImageJ to demark DAPI-stained regions) within 8 h of HU release and <2-fold increase at 24 h. Interestingly, both DNA-PKcs null and KD mutant cells showed significant increases in average size of nuclei at early times, increasing >3-fold at 24 h (Figure 6C). The persistent high γ-H2AX signal and increasing nuclear size after HU release in DNA-PKcs mutant cells was not due to increased amounts of DNA as DAPI signals did not change over time (Figure 6D). As DNA-PKcs plays an important role in phosphorylating RPA32 Ser4/Ser4 in response to replication stress, we tested whether RPA32 S4A/S8A mutant cells would also show persistent damage (γ-H2AX) after release from stress. Similar to DNA-PKcs mutant cells, S4A/S8A mutant cells showed more persistent γ-H2AX signals after release from etoposide, assayed by immunofluorescence microscopy (Figure 7A and B) and flow cytometry (Figure 7C). These results indicate that DNA-PKcs does not prevent replication fork collapse during HU treatment, but it does prevent the accumulation of damage after HU release, probably reflecting its role in maintaining replication checkpoint arrest.

### RPA32 phosphorylation prevents mitotic progression of cells with DNA damage

**Figure 8.**
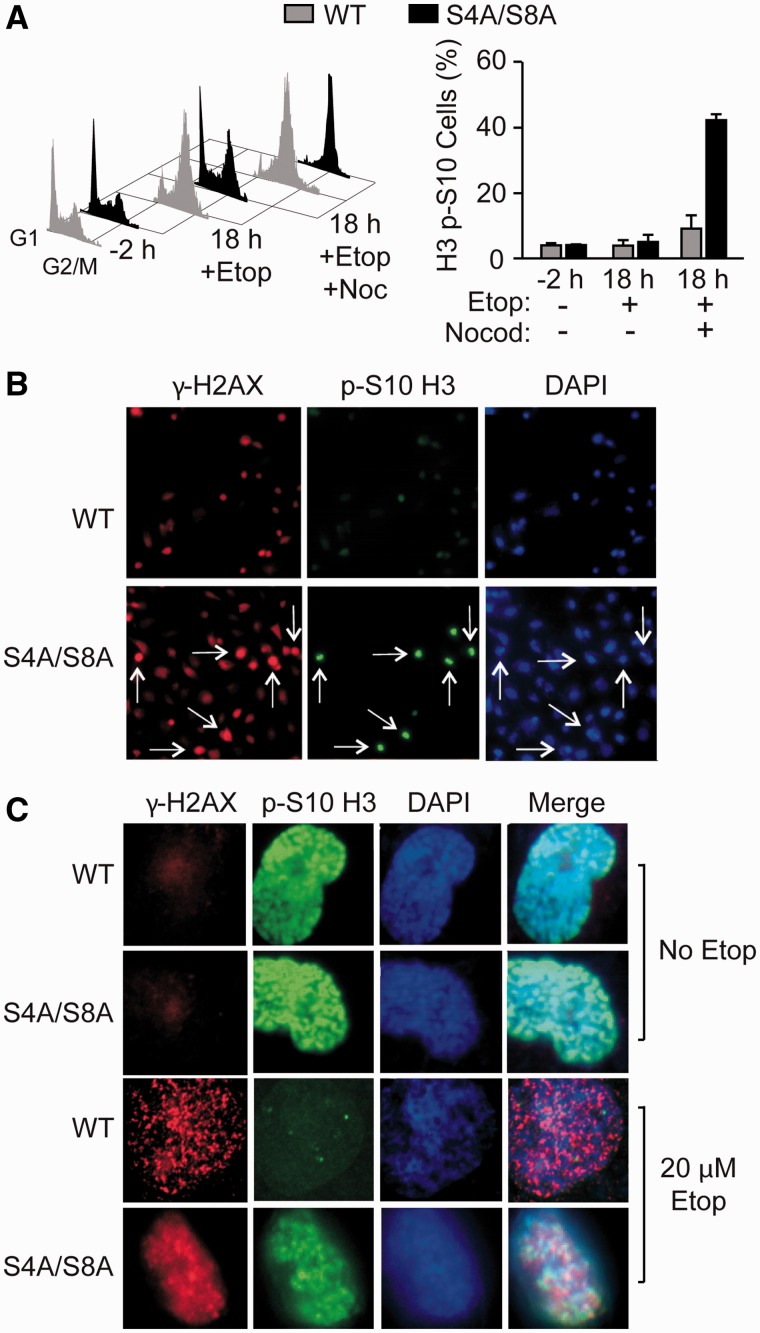
Phosphorylation of RPA32 Ser4/Ser8 promotes etoposide-induced S and G2/M checkpoint arrest. (**A**) Cell cycle profiles (DNA content) in WT and S4A/S8A RPA32 cells treated with etoposide for 2 h and released, or trapped in M phase with nocodazole. Graphed are the average percentage M phase cells (±S.E.M. for three independent experiments) determined by flow cytometry analysis of immunofluorescent p-S10 H3 stained cells. (**B**) Representative immunofluorescence images of γ-H2AX, p-S10 H3 and DAPI stained nuclei in WT and S4A/S8A RPA32 cells treated with etoposide for 2 h and released for 18 h. (**C**) High magnification of nuclear staining patterns from panel B, and including images of mitotic cells from untreated control cultures.

Cells are particularly vulnerable to genome destabilizing and cytotoxic effects of DNA damage when DNA is replicated in S phase and during chromosome segregation in M phase. The Chk1 activation defect and persistent γ-H2AX seen with mutant DNA-PKcs or S4A/S8A RPA32 suggests that DNA damage accumulates when these cells fail to arrest in S phase in response to replication stress. To determine if RPA32 Ser4/Ser8 phosphorylation is also important to prevent damaged cells from progressing into M phase, we treated cells with etoposide and analyzed WT and S4A/S8A mutant cell cycle profiles and phosphorylation of histone H3 Ser10 (pS10-H3), an M phase marker. Etoposide causes WT cells to accumulate in S phase, but the cell cycle profile of S4A/S8A-RPA32 mutant cells are nearly identical in the presence or absence of etoposide. This raised the question of whether these cells were actively cycling through the cell cycle, or had become cytostatic (59). We used the mitotic inhibitor nocodazole to trap cells in mitosis to test checkpoint integrity and dynamic cell cycle progression. Upon etoposide and nocodazole treatment, cells expressing S4A/S8A-RPA32 accumulated in M phase, indicating that they were progressing past the G2/M boundary, and we observed 4-fold more pS10-H3 positive cells than WT (Figure 8A). Taken together, these data indicate that S4A/S8A-RPA32 lack S and G2/M checkpoint arrest, allowing cells to continue into mitosis.

As DNA-PKcs and S4A/S8A mutant cells display what appears to be persistent DNA damage (γ-H2AX) and progress inappropriately into M phase after replication stress, we asked whether damaged cells progress into M phase. We treated cells with 20 µM etoposide for 2 h, and 18 h later, analyzed pS10-H3 and γ-H2AX using immunofluorescence microscopy. At low magnification, WT cells with γ-H2AX foci contained only background levels of pS10-H3 (Figure 8B), indicating that damaged cells were not progressing into mitosis. In contrast, many cells expressing S4A/S8A-RPA32 that were positive for γ-H2AX were also positive for pS10-H3, indicating that these mutant cells were progressing into mitosis with damaged DNA (Figure 8B). Higher magnification revealed additional detail in the staining patterns (Figure 8C). In the absence of etoposide, cells positive for phosphorylated H3 staining were nearly devoid of γ-H2AX in WT and S4A/S8A mutant cells. After etoposide treatment, WT cells showed punctate γ-H2AX foci with background levels of pS10-H3, and S4A/S8A mutant cells showed pan-nuclear γ-H2AX and pS10-H3 staining. These results indicate that defective G2/M checkpoint activation in cells expressing S4A/S8A-RPA32 allows inappropriate mitotic entry in the presence of unresolved DNA damage.

## DISCUSSION

Replication stress-induced RPA32 phosphorylation shows complex patterns of PIKK targeting and phosphorylation site priming effects. Anantha *et al.* (29) showed that Thr21 phosphorylation primes Ser4/Ser8 phosphorylation, and here we show that phospho-Ser4/Ser8 is almost essential for Thr21 phosphorylation (Figure 1C). Thus, these sites show partial reciprocal priming effects, with phospho-Ser4/Ser8 showing a stronger effect. This contrasts with Ser33 and Ser4/Ser8, as Ser33 primes Ser4/Ser8 (29,31), but not *vice versa* (Figure 2D).

ATR plays a prominent role in replication stress responses, in part through phosphorylation of RPA32. RPA32 Ser33 is a well-established ATR target that primes phosphorylation of Ser29 and Thr21 (29). We confirmed the ATR-dependent phosphorylation of Ser33, but unlike Thr21, Ser33 phosphorylation occurs independently of Ser4/Ser8 phosphorylation. Liaw *et al.* (35) established that DNA-PKcs phosphorylates Ser4/Ser8 *in vivo*. *In vitro*, we found that Ser4/Ser8 is not phosphorylated by ATR, it is weakly phosphorylated by ATM, and it is strongly phosphorylated by DNA-PKcs (Figure 2). Our *in vivo* results with ATMi and DNA-PKi are consistent with these *in vitro* findings (Figure 2D). These results suggest that DNA-PK is the primary kinase targeting Ser4/Ser8 after replication stress, with ATM playing a lesser role.

Ser12 is also targeted by DNA-PK and ATM, but Thr21 appears to be largely or exclusively phosphorylated by DNA-PK (Figure 2). The Thr21 result contrasts with Block *et al.* (20), who reported that Thr21 is phosphorylated normally in response to etoposide in DNA-PKcs mutant human M059J cells, and in ATM-defective cells. This group also reported that Thr21 phosphorylation was blocked in the ATM-defective cells by the DNA-PKcs and ATM inhibitors wortmannin and caffeine, implicating ATR. Our results are consistent with little or no role for ATM, but how can the DNA-PKcs discrepancy be explained? Block *et al.* (20) showed that Thr21 is phosphorylated by DNA-PKcs *in vitro*, consistent with our results (Figure 2A–C), and their *in vivo* result also contrasts with Anantha *et al.* (29) who showed that Thr21 phosphorylation was reduced, but not eliminated in M059J cells. A possible explanation is that in the absence of DNA-PKcs (in M059J), ATR plays a larger role in Thr21 phosphorylation, but when DNA-PKcs is present but inhibited, it prevents ATR from targeting Thr21, analogous to the situation seen with H2AX phosphorylation by ATM and DNA-PKcs, as discussed by Stucki and Jackson (60). It is also possible that the wortmannin/caffeine result reflects priming effects, as ATR phosphorylates Ser33 which primes Ser4/Ser8 and Ser29 (29) and Ser4/Ser8 phosphorylation primes Thr21 (Figure 2D). It is noteworthy that human DNA-PKcs knock-out cells are nearly inviable (61), suggesting that M059J cells harbor compensating mutations beyond those known in p53 and ATM (62,63).

DNA-PKcs is activated when it binds to Ku-bound DNA ends at DSBs (64), Block *et al.* (20) argued that DNA-PKcs cannot be activated in response to etoposide-induced DNA damage because etoposide traps TopoIIα covalently bound to DNA ends, preventing access of Ku and DNA-PKcs, and this argument was supported by *in vitro* analysis of DNA-PKcs activation (65). However, there is evidence that DNA-PKcs can act independently of Ku, and at single-strand breaks (66,67). In addition, Block *et al.* treated cells with etoposide for 2 h, then immediately lysed the cells for RPA32 analysis by immunoprecipitation and western blot. Although RPA32 phosphorylation is apparent after a 2-h etoposide treatment, in our experience, the signal continues to increase for several hours after release from etoposide (S. Liu *et al.*, unpublished results), suggesting that DNA-PKcs may not be activated by the initial TopoIIα–DNA complex but sometime later when these lesions are processed, e.g. by MRN or xeroderma pigmentosum G (XPG) or TopoIIα proteolysis, and/or stalled forks collapse to DSEs, which then enter the RPA-ATRIP-ATR pathway. The degree of RPA phosphorylation also likely depends on the type of replication stress. CPT is a TopoI inhibitor that induces RPA phosphorylation by DNA-PKcs very rapidly, within 30 min (19). As TopoI plays a key role upstream of replication forks, CPT may create greater amounts of ssDNA than etoposide. This ssDNA would be efficiently bound by RPA, activating ATR, which would phosphorylate RPA32 Ser33 which then primes phosphorylation of other N-terminal residues (68). Thus, DNA-PKcs phosphorylation of RPA32 may show temporal variations depending on the type of replication stress.

PIKKs phosphorylate proteins at S/TQ consensus sites. RPA32 Thr21 is a consensus PIKK site, but this study and others indicate that DNA-PKcs also targets non-consensus sites in RPA32 including Ser4, Ser8 and Ser12, as seen in other DNA repair proteins including Artemis, XRCC4 and Ku (69–71). The sequences surrounding Ser4, Ser 8 and Ser12 - (MWN**S**GFE**S**YGS**S**S) include hydrophobic, negatively charged and other Ser residues, which are known to enhance substrate recognition by DNA-PK (72).

**Figure 9.**
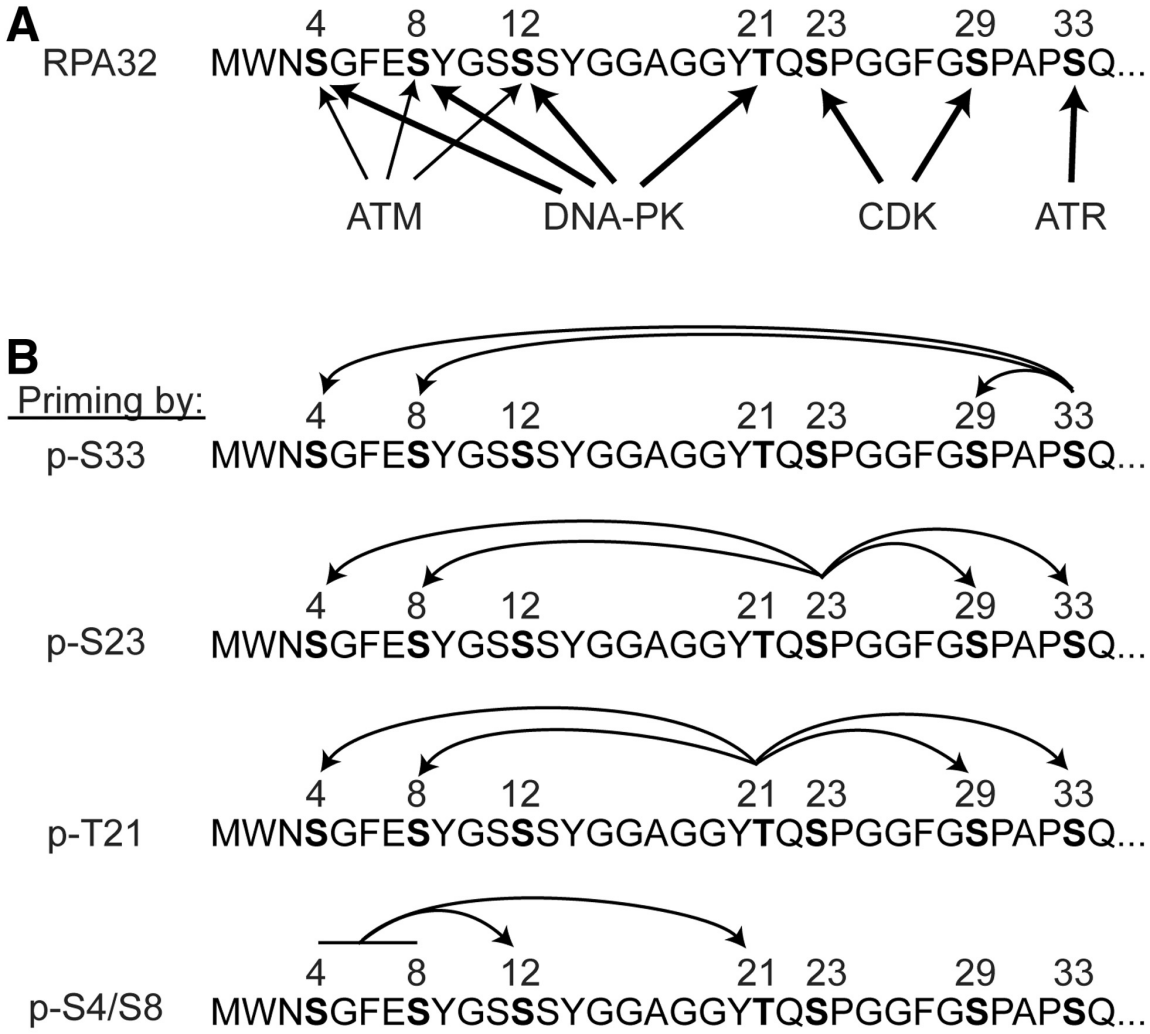
RPA32 phosphorylation by PIKKs/CDK. (**A**) Each PIKK and CDK targets a subset of RPA32 residues. Dominant activities are shown with thick arrows, minor activities by thin arrows. There is likely more redundancy than is shown, and different replication stress agents are likely to induce different PIKK/CDK targeting patterns. (**B**) Priming patterns for specific RPA32 phospho-residues. Patterns for Ser33, Ser23 and Thr21 were determined previously (29,31), and for Ser4/Ser8 in this study (Figures 1 and 2).

The results of this study and previous studies (20,68) indicate that each PIKK phosphorylates distinct sets of RPA residues with variable efficiencies in response to replication stress. Our results extend previous observations of RPA phosphorylation priming (29,31), demonstrating that through phospho-Ser4/Ser8 priming effects, DNA-PK regulates phosphorylation of Ser12, Thr21 and Ser33 (Figures 1C and 2D). The complex PIKK targeting and RPA phospho-priming relationships are summarized in Figure 9. Given that Ser4/Ser8 phosphorylation marks the mature, hyperphosphorylated form of RPA32, which is required for replication checkpoint arrest (30,32–34), our results indicate that along with ATR phosphorylation of Ser33, DNA-PKcs phosphorylation of Ser4/Ser8 and Thr21 plays a key role in RPA32-dependent replication checkpoint signaling (discussed further below).

Despite early evidence that RPA phosphorylation was not required for checkpoint activation (73), evidence from the present study and previous studies indicates otherwise (19,29,31,35,74,75). For example, RPA32 hyperphosphorylation and phospho-Ser4/Ser8 in particular, are important for upstream checkpoint signaling through phosphorylation/activation of MRE11 and TopBP1 (Figure 3B and C), and ultimately, Chk1 activation (Figure 4). Ser4/Ser8 phosphorylation is not important for the Chk2 arm of the DNA damage response (Figure 3A). Interestingly, Ser4/Ser8, Thr21 and Ser33 are phosphorylated rapidly after replication stress and correlate with replication arrest, but Ser12 is phosphorylated late when the replication checkpoint is inactivated and the replication resumes (Figure 5).

It has been proposed that PIKK phosphorylation of RPA switches RPA from its normal replication mode to a repair mode (31,68,76). Although this concept is appealing, mechanistic details are lacking. Here, we show that defects in RPA32 hyperphosphorylation, either through S4A/S8A mutation or DNA-PKcs defect, lead to increased levels of collapsed replication forks, marked by γ-H2AX and that these signals are more persistent (Figures 6 and 7). The fact that cells bearing DNA-PKcs or RPA32 S4A/S8A mutations show a similar γ-H2AX phenotype when subjected to replication stress further supports the idea that DNA-PKcs is the major kinase targeting Ser4/Ser8 in response to stress. In addition to its replication checkpoint defect, the S4A/S8A mutant also fails to arrest at the G2/M border, and cells bearing significant damage (γ-H2AX) enter mitosis (Figure 8). In a separate study, we explore in more detail the genetic consequences of failed checkpoint arrest due to defects in PIKK phosphorylation of RPA32 in response to replication stress (manuscript in preparation).

In summary, PIKK phosphorylation of RPA32 plays a key role in replication checkpoint activation, and DNA-PK has emerged as an important contributor to this response. DNA-PK has long been known for its roles in DSB repair by NHEJ, and its ∼20-fold greater abundance in human cells than rodent cells has fueled research that has uncovered several DNA repair-independent roles including gene regulation and proliferation homeostasis (77). Through the complex RPA32 phosphorylation priming relationships, including reciprocal priming, DNA-PK now emerges as an equal partner with ATR in the S phase checkpoint response, regulating key downstream events including MRE11 and TopBP1 phosphorylation and Chk1 phosphorylation/activation via targeting of RPA32 Ser4/Ser8 and Thr21. As with other NHEJ proteins, DNA-PKcs has long been considered a target to sensitize tumor cells to radiation therapy, leading to the development of specific DNA-PKcs inhibitors such as DMNB and NU7026. In light of its critical role in replication checkpoint activation, such inhibitors may prove valuable when paired with classical cancer chemotherapeutics, like etoposide and CPT that induce replication stress.

## FUNDING

The National Institutes of Health [P20RR018759-08 to G.G.O. and R01 CA100862 and R01 GM084020 to J.A.N.]; American Cancer Society [RSG-10-031-01-CCG to G.G.O.]; Department of Human and Health Services of Nebraska (to G.G.O.). Funding for open access charge: College of Dentistry Research Fund and the Department of Oral Biology, UNMC.

*Conflict of interest statement*. None declared.
